# Effects of esketamine on depression-like behavior and dendritic spine plasticity in the prefrontal cortex neurons of spared nerve injury-induced depressed mice

**DOI:** 10.1590/1414-431X2024e13736

**Published:** 2024-07-08

**Authors:** Bixin Huang, Xiaoling Li, Yuling Zheng, Ying Mai, Zhongqi Zhang

**Affiliations:** 1 Department of Anesthesiology, The Affiliated Shunde Hospital of Jinan University, Foshan, Guangdong, China

**Keywords:** Esketamine, Depression, CRMP2, Spared nerve injury, Dendritic spine, Prefrontal cortex

## Abstract

The present study utilized the spared nerve injury (SNI) to create a mouse model of depression to investigate the impact of esketamine on depressive-like behaviors, on the expression of PSD-95 and CRMP2 proteins, and on changes in neuronal dendritic spine plasticity in the prefrontal cortex (PFC). Depressive-like behavioral tests were performed 1 h after esketamine treatment, and the PFC tissues were obtained on the fourth day after completing the behavioral tests. Then, dendritic spine density and morphology in the PFC were measured using Golgi staining, and CRMP2 and PSD-95 proteins were obtained from PFC tissue by western blotting. The results of this study showed that esketamine significantly increased the immobility time in the forced swimming test and tail suspension test. In the open field test, esketamine increased the time spent in the open arms, the time spent in the central area, and the total distance covered. It also increased the protein expression levels of CRMP2 and PSD-95 in addition to the total and mature dendritic spine density of the PFC in SNI-depressed mice. Esketamine can significantly improve depression-like behaviors in SNI-depressed mice and promote an increase in dendritic spine density and maturation in the PFC. These effects may be associated with changes in CRMP2 and PSD-95 expression.

## Introduction

Emotional disorders are commonly associated with chronic pain, which is a significant health problem affecting numerous individuals. Long-term pain can cause various negative changes in the body and mind, including the development of negative emotions and behaviors such as depression and anxiety (1,2). Research has demonstrated a strong correlation between depression and pain, indicating that approximately 20-50% of individuals experiencing chronic pain also suffer from depression. Moreover, the prevalence of depression tends to be higher among patients coping with intense pain levels (3,4).

The spared nerve injury (SNI) model is extensively utilized to investigate chronic pain and accompanying depressive-like behaviors. A sciatic nerve injury can lead to the development of chronic pain, which may trigger depressive-like behavior (5). Currently, numerous methods exist for treating depression, but satisfactory outcomes have not yet been achieved.

Antidepressant medications may improve depressive symptoms by regulating synaptic plasticity and promoting the re-establishment of neural circuits (6). Esketamine has been widely used in depression treatment and has two enantiomers that are mirror images of each other: (S)-enantiomer and (R)-enantiomer. Esketamine is an (S)-enantiomer belonging to a new class of rapidly acting antidepressants. It was approved by the United States Food and Drug Administration (FDA) in 2019 for treating resistant depression (7). It is a non-competitive N-methyl-D-aspartate (NMDA) receptor antagonist that regulates the glutamate system, modulates neural transmission processes, and exerts a rapid antidepressant effect (8).

The prefrontal cortex (PFC) is an important brain region that regulates emotional and cognitive functions. Depression can lead to structural and functional abnormalities in the PFC (9). Plastic changes in the dendritic spines of neurons play an important role in regulating PFC function (10), and depression may be closely related to the atrophy of PFC neurons and the reduction of synapses. Esketamine administration can induce rapid synaptogenesis within hours (11). However, its specific mechanism remains unclear. Many studies have revealed the significant role of postsynaptic density protein-95 (PSD-95) in depressed mice, and depressed mice display downregulated PSD-95, which is promptly upregulated following treatment with esketamine and is linked to the augmentation of dendritic spine density in the PFC (6,12,13). Collapsin response mediator protein 2 (CRMP2) is crucial in neuronal growth and development. Our preliminary study has discovered that ketamine administration can significantly enhance the density and maturity of dendritic spines in newborn mouse PFC neurons, primarily through Cdk5 phosphorylation-mediated activation of CRMP2 (14). While there has been extensive research on the association between esketamine and dendritic spines in the PFC of depressed mice and its correlation with depressive-like behaviors, studies in SNI-depressed mice are rare, and the specific mechanism is not yet fully understood.

Therefore, this study utilized the SNI to create a mouse model of depression to investigate the impact of esketamine on depressive-like behaviors, the expression of PSD-95 and CRMP2 proteins, and the changes in neuronal dendritic spine plasticity in the PFC. This research will contribute to improve our understanding of the molecular mechanisms that underlie the antidepressant properties of esketamine.

## Material and Methods

### Animals

**Figure 1:**
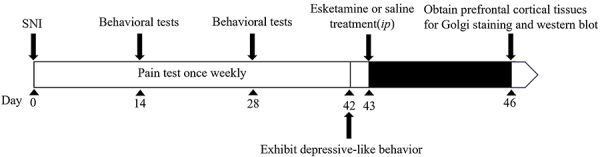
Experimental timeline. The spared nerve injury (SNI) model was established in mice from day 1 to 42. On day 42, mice exhibited depressive-like behaviors. The mice were acclimated to the testing environment for one day. On day 43, esketamine (10 mg/kg) or an equal volume of saline was intraperitoneally (*ip*) administered to the SNI-depressed or sham mice. The 4 behavioral tests, which included the open field test (OFT), elevated plus maze (EMP), forced swim test (FST), and tail suspension test (TST), were conducted one hour later. On day 46, prefrontal cortical tissues were obtained from the mice for Golgi staining and western blotting analysis.

Male C57BL/6 mice weighing between 20-24 g and aged 8-9 weeks were acquired from Liaoning Changsheng Biotechnology Co., Ltd. (China). They were housed in a temperature-controlled room with a temperature range of 20-25°C and humidity level of 40-60%. The light/dark cycle was set to 12 h (from 8:00 to 20:00). The animal experiments were conducted following the “Regulations of the People's Republic of China for the Administration of Laboratory Animals” and approved by the “Animal Ethics Committee of Jinan University (China)”, in compliance with the regulations on the management of experimental animals. The timeline of the experimental design is illustrated in Figure 1.

### Establishment of the SNI model

We referred to a previous study to guide the construction of an SNI neuropathic pain model (15). All mice were anesthetized with 2% isoflurane and underwent ligation of the common peroneal nerve and tibial nerve using an 8-0 nylon thread at the distal end of the sciatic nerve while ensuring the integrity of the sural nerve and avoiding any stretching or damage. The sham-operated mice served as controls and only underwent exposure of the sciatic nerve branches. Muscle and skin suturing were performed using a 4-0 nylon thread, and the sutured area was disinfected.

### Pain test and drug administration

The mice were placed in a transparent glass box and allowed to acclimatize for 45 min. The Von Frey test was employed to evaluate the postoperative occurrence and maintenance of mechanical abnormal pain once weekly (16). The study was divided into three groups: Sham-control (Con group), SNI-Saline (Saline group), and SNI-esketamine (Esk group). The treatment methods for the three groups were as follows: 1) Con group: sham-operated mice received an intraperitoneal injection of an equal volume of saline; 2) Saline group: depressed mice received an intraperitoneal injection of an equal volume of saline; 3) Esk group: depressed mice were given a single intraperitoneal injection of 10 mg/kg esketamine (2 mL/50 mg, Jiangsu Hengrui Medicine, China, lot number: 230623BL; esketamine was diluted with 0.9% NaCl to a constant volume 0.2 mL in all mice).

### Depressive-like behavioral tests

Behavioral tests were performed at the 2nd, 4th, and 6th weeks post-surgery. If depressive-like behaviors were observed, drug treatment was administered the following day. One hour after drug administration, behavioral tests commenced. Additionally, mice were placed in the testing environment 45 min prior to each experiment to acclimate.

Between each behavioral test, the experimental equipment was wiped with 75% alcohol to avoid the influence of odors from other mice on the experimental results. Video tracking systems were employed to record the behavior of the animals during testing. All behavioral tests were automatically analyzed using Topscan Lite (Version 2.0, CleverSys Inc., USA).

#### Forced swimming test

A forced swimming test (FST) was conducted according to a previous report (17). The main apparatus for the experiment was a cylindrical water bucket with a height of 28 cm and a bottom diameter of 16 cm. Before the experiment, the bucket was filled with water to a height of approximately 20 cm, and the water temperature was maintained at approximately 20-21°C. Once the experiment commenced, mice were removed from their cages and placed in the water. The duration of the experiment was 6 min. Subsequently, the total immobility time of the mice in the water in the last 4 min was recorded.

#### Tail suspension test

Tail suspension test (TST) was conducted following the established protocol (18). At the beginning of the experiment, tape was wrapped around the tail of the mice at approximately 1.5 cm from the tip. The mice were then suspended in a box of 20×20×30 cm. The duration of the entire experiment was 6 min. Subsequently, the immobility time of the mice in the last 4 min was recorded.

#### Elevated plus maze

The elevated plus maze (EPM) test was conducted following established procedures (19). The elevated plus maze is a cross-shaped maze 50 cm above the ground, consisting of four arms and a square center area (5×5 cm). The four arms consist of two open arms (30×5×15 cm) and two closed arms. Mice were taken out of their cages and placed in the center of the maze at the beginning of the experiment. They were then given 10 min to freely explore the maze. Subsequently, the number of entries into the open arms and the total time spent in the open arms were recorded.

#### Open field test

Open field test (OFT) was performed as previously described (20,21). Mice were removed from their cages and placed individually in the center of an empty box (40×40×40 cm). Mice were allowed to freely explore the empty box for 10 min. Subsequently, the total time spent in the central area and the total distance covered by the mice throughout the box were recorded.

### Golgi staining and western blotting

The mice were euthanized on the fourth day after the completion of the behavioral tests. The brains were dissected to obtain PFC tissues. The surfaces of the tissues, blood, and other components were cleaned using cooled phosphate-buffered saline (PBS) or double-distilled water. Golgi staining was employed as in our previous research methods (14). The FD Rapid Golgi Stain Kit (FD Neuro Technologies, USA) was utilized for Golgi staining. Coronal sections (100 μm) were obtained using a Leica Vibratome (VT1000S, Germany). Western blotting was performed according to our previous research protocol (14). Total protein samples were obtained from the PFC tissue, and their concentrations were measured using BCA assay. The protein was loaded onto a gel for electrophoresis, transferred onto a membrane, sealed, incubated with the primary antibody (anti-CRMP2 antibody and anti-PSD-95 antibody, concentration 1:1000, Cell Signaling Technology, USA) overnight, and incubated again with the secondary antibody (anti-rabbit IgG, diluted at 1:2000, Jackson, USA). The membrane was blocked with 5% non-fat milk and incubated for 1 h at room temperature. β-actin was used as the loading control. The band intensities were analyzed using Quantity One software (BIO-RAD, USA) following the manufacturer's instructions. The western blotting results presented here were obtained from three separate and rigorously equal experiments.

### Statistical analysis

The study utilized the GraphPad Prism 9.0 (USA) software for generating graphs and conducting statistical analyses. The experimental data are reported as means±SE. One-way analysis of variance (ANOVA) was performed to compare and analyze differences among multiple groups. P<0.05 denoted statistical significance.

## Results

### Effects of esketamine on the depressive-like behaviors of SNI-induced depressed mice

**Figure 2:**
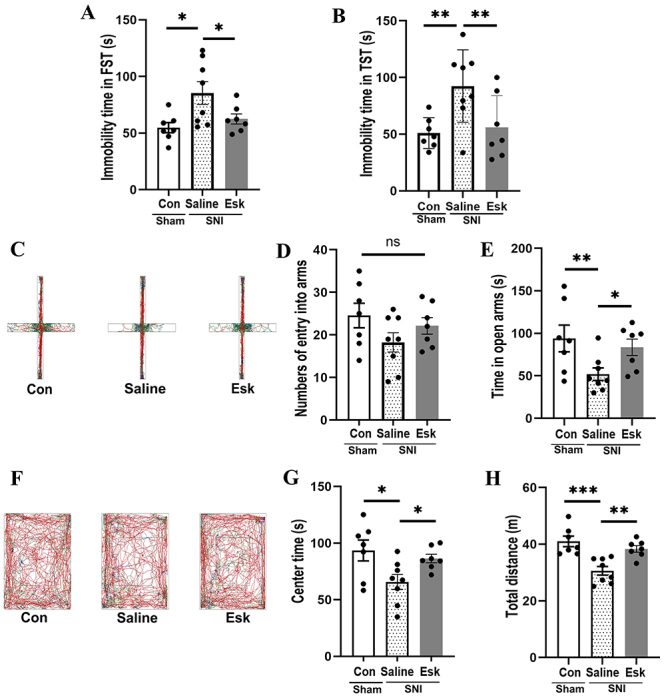
Effects of esketamine in depressive-like behavior of the three groups. **A**, Time of immobility during the forced swimming test (FST) (F=4.979, P=0.018). **B**, Time of immobility during the tail suspension test (TST) (F=5.729.721, P=0.011). **C**, Movement trials in the elevated plus maze (EPM); **D**, Number of entries into the open arms during EPM (F=1.817, P=0.190). **E**, Time spent in the open arms during EPM (F=3.994, P=0.036). **F**, Movement trails in the open field test (OFT). **G**, Amount of time spent in the central area during the OFT (F=4.532, P=0.025). **H**, Total distance traveled during the OFT (F=17.368, P=0.000). Con (control) group (n=7), Saline group (n=8), and Esk (spared nerve injury-esketamine) group (n=7). Data are reported as means±SE. *P<0.05, **P<0.01, ***P<0.001 (ANOVA), ns: no significant difference.

Depressive-like behaviors appeared in SNI mice on the 42nd day after surgery. Compared with the Saline group, the immobility time in the FST was significantly increased in Con and Esk groups (P<0.05). Additionally, the immobility time in the TST significantly increased (P<0.01). No statistical difference was identified in the immobility time in the FST and TST between Con and Esk groups (Figure 2A and B).

In the EPM, there were no significant differences in the number of entries into the open arms among the three groups of mice. However, the duration of stay in the open arms was significantly decreased in the Saline group compared to the Con and Esk groups (P<0.05). No statistical difference was observed in the duration of stay in the open arms between Con and Esk groups (Figure 2C, D, and E).

In the OFT, compared with the Saline group, the time spent in the central area and total distance covered in Con and Esk groups were significantly increased (P<0.05). No statistical difference was observed in time spent in the central area and total distance moved between Con and Esk groups (Figure 2F, G, and H).

From the above behavioral experiments, esketamine caused a significant improvement in the depression-like behaviors of SNI-induced depressed mice.

### Effects of esketamine on the expression of CRMP2 and PSD-95 proteins in the PFC of SNI-induced depressed mice

**Figure 3:**
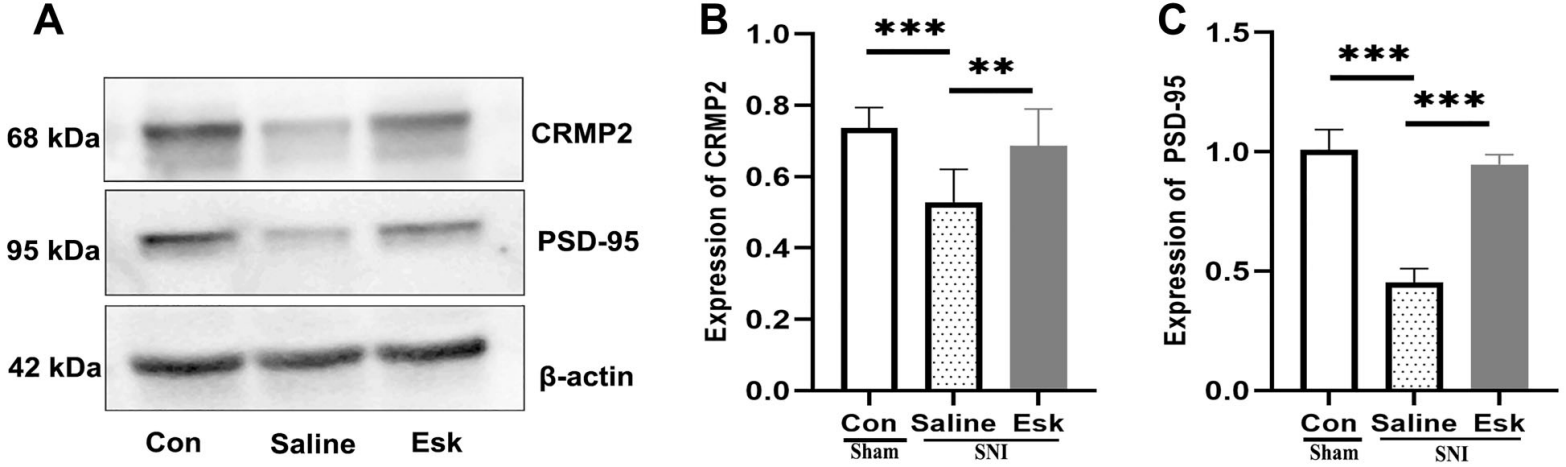
Protein expression of collapsin response mediator protein 2 (CRMP2) and postsynaptic density protein-95 (PSD-95) in the prefrontal cortex (PFC) of the three groups. **A**, Western blotting bands of CRMP2 and PSD-95. **B**, Expression of CRMP2 protein (F=12.180, P=0.000). **C**, Expression of PSD-95 protein (F=173.180, P=0.000). At least three independent western blot experiments were performed for each group. Data are reported as means±SE. **P<0.01, ***P<0.001 (ANOVA). Con: control group; Esk: spared nerve injury-esketamine.

Compared with the Con group, the protein expression levels of CRMP2 and PSD-95 in the PFC tissue of the Saline group were significantly decreased (P<0.001). After esketamine administration, the expression levels showed a significant increase (Saline group *vs* Esk group, P<0.01). No statistical difference was found between Con and Esk groups (Figure 3).

### Effects of esketamine on dendritic spine development in PFC neurons of SNI-induced depressed mice

**Figure 4:**
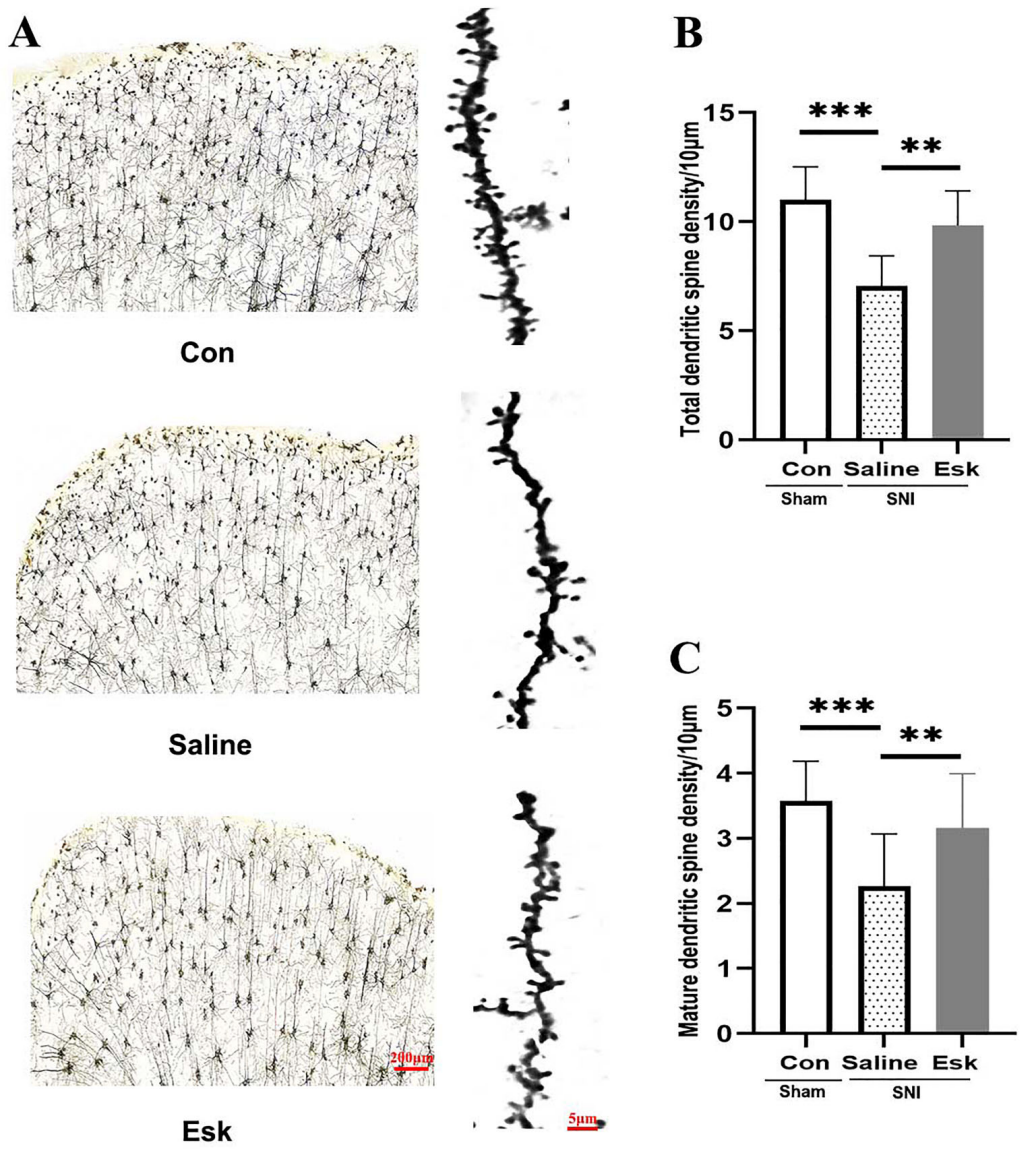
Golgi staining in the prefrontal cortex (PFC) of the three groups. **A**, PFC neurons and dendritic spines. Scale bars, 200 μm and 50 μm. **B**, Total dendritic spine density (F=38.769, P=0.000). **C**, Mature dendritic spine density (F=15.020, P=0.000). Three independent Golgi staining experiments were conducted for each group, and 20 neuronal dendrites were counted in the PFC per group. Data are reported as means±SE. **P<0.01, ***P<0.001 (ANOVA). Con: control group; Esk: spared nerve injury-esketamine.

Dendritic spine morphology includes several types: mushroom-shaped, stubby, thin and long, and filopodia. Stubby and mushroom-shaped are mature forms, whereas thin, long, and filopodia spines indicate immature states (22,23). Golgi staining results (Figure 4A) showed that the total dendritic spine density of PFC neurons in Con and Esk groups was significantly higher than that of the Saline group (P<0.01), without a statistical difference between Con and Esk groups (Figure 4B). In terms of mature dendritic spine density, Con and Esk groups exhibited a significant increase compared to the Saline group (P<0.01), without a statistical difference between Con and Esk groups (Figure 4C).

Golgi staining results indicated that esketamine significantly increased the total dendritic spine density and maturation in PFC neurons of SNI-induced depressed mice.

## Discussion

Depression is a prevalent psychiatric condition that seriously threatens the physical and mental health of individuals. Depression development involves genetic and acquired factors, with chronic pain being the prominent factor. Chronic pain, when endured for an extended period, not only diminishes the quality of life but also intensifies negative emotions and psychological stress, thereby precipitating or worsening depressive symptoms (24-26). The SNI model is a reliable means of inducing depression associated with neuropathic pain (27,28). Our study revealed that mice displayed symptoms resembling depression after six weeks. In this study, esketamine, a fast and effective antidepressant drug, was administered intraperitoneally to observe its effects on depression-like behavior, PSD-95 and CRMP2 protein expression, and dendritic spine plasticity in PFC neurons. The findings indicated a significant improvement in depression-like behavior in mice treated with esketamine, accompanied by elevated expression of PSD-95 and CRMP2 proteins and enhanced dendritic spine density and maturity in PFC neurons.

Despite drawbacks such as slow reaction times, significant individual differences, long-term usage issues, and multiple adverse reactions, pharmacotherapy remains a commonly utilized treatment method for depression (29,30). In this study, we administered esketamine via an intraperitoneal injection to depressed mice. Subsequently, depression-related behavioral tests were performed after 1 h. The results showed significant increases (P<0.05) in immobility time in the FST and TST and time spent in the open arms, time spent in the central area, and total distance traveled in the OFT after esketamine administration compared with the depressed group. These findings suggested that esketamine has rapid and effective antidepressant effects.

Dendritic spines are small protrusions with branches on the cell bodies of neurons, and their morphology and function are influenced by external stimuli and internal signals. The forming of dendritic spines in the PFC plays a crucial role in maintaining specific antidepressant effects and long-term relief (6). The number and morphology of dendritic spines in PFC neurons of depressed rats undergo changes, primarily manifested by a decrease in mature dendritic spines and an increase in immature dendritic spines, thereby resulting in significant effects on synaptic function and receptor expression (31). A study observed a marked reduction in dendritic spine density in PFC neurons of mice with depression, which may be linked to decreased memory and learning abilities, as well as impaired decision-making abilities (32). Moreover, alterations in the dendritic spine morphology of neurons in the PFC encompass aberrations in dendritic spine structure and branching patterns (33). In this study, we performed Golgi staining on the PFC three days after intraperitoneal injection of 10 mg/kg esketamine. We found a marked decrease in dendritic spine density in the PFC of depressed mice, particularly in mature dendritic spines. These findings suggested that alterations in mature dendritic spines may result in decreased interconnections among PFC neurons, ultimately affecting normal brain function (23). This also indicated that improved depression symptoms by esketamine may be achieved by altering the plasticity of dendritic spines in PFC neurons. However, the specific mechanisms involved require further investigation.

CRMP2 and PSD-95 are highly expressed during central nervous system development. A study revealed that sevoflurane induces dendritic developmental abnormalities and cognitive dysfunction in rats, primarily through the phosphorylation of CRMP2 protein in nerve cells induced by sevoflurane (34). This process was accompanied by a decrease in PSD-95 protein expression. By studying CRMP2 gene knockout mice, Zhang et al. (35) discovered a substantial reduction in the total length of dendrites, in the complexity of neuronal dendrites, and in the density of dendritic spines. In addition, electron microscopy revealed a significant reduction in PSD thickness. These studies indicate that CRMP2 and PSD-95 play important roles in developing dendrites and synapses in the neurons. The present study revealed that the expression levels of CRMP2 and PSD-95 proteins were significantly reduced in the PFC of SNI-induced depressed mice. However, esketamine administration significantly increased the expression levels of CRMP2 and PSD-95 proteins, suggesting a potential role of CRMP2 and PSD-95 in depression pathogenesis.

The limitations of this study were as follows: 1) This study used SNI-induced depressed mice to observe the effects of esketamine on the expression of CRMP2 and PSD-95 proteins without further investigation of the functions and regulatory mechanisms of these proteins. Additional research is necessary to achieve a comprehensive understanding of the pathogenesis of depression and provide new targets for related treatment methods. 2) Multiple studies have shown that rodents with depression display comparable alterations in the dendritic spine structure of neurons in the hippocampus and PFC (36-38). However, this study did not investigate alterations in dendritic spine morphology in the hippocampal neurons. 3) Although we used a comprehensive suite of behavioral tests to assess depression-like behaviors in SNI-induced depressed mice, this study did not include the sucrose preference test (SPT). The SPT is widely recognized as a critical measure for evaluating anhedonia, as depressed mice exhibit diminished interest or pleasure in sweet stimuli (39). Future studies could benefit from including the SPT to provide a more holistic understanding of the antidepressant effects of esketamine.

In summary, a single intraperitoneal injection of esketamine at a dose of 10 mg/kg significantly improved depressive-like behaviors in SNI-depressed mice and promoted increased dendritic spine density and maturation in the PFC. These effects may be associated with changes in CRMP2 and PSD-95 expression.
